# Towards the characterization of the tumor microenvironment through dictionary learning-based interpretable classification of multiplexed immunofluorescence images

**DOI:** 10.1088/1361-6560/aca86a

**Published:** 2022-12-21

**Authors:** Santhoshi N Krishnan, Souptik Barua, Timothy L Frankel, Arvind Rao

**Affiliations:** 1 Department of Electrical and Computer Engineering, Rice University, Houston, Texas-77005, United States of America; 2 Department of Computational Medicine and Bioinformatics, University of Michigan, Ann Arbor, Michigan-48109, United States of America; 3 Department of Surgery, University of Michigan, Ann Arbor, Michigan-48109, United States of America; 4 Department of Biostatistics, University of Michigan, Ann Arbor, Michigan-48109, United States of America; 5 Department of Biomedical Engineering, University of Michigan, Ann Arbor, Michigan-48109, United States of America; 6 Department of Radiology, University of Michigan, Ann Arbor, Michigan-48109, United States of America

**Keywords:** dictionary learning, immunotherapy, pancreatic malignancies, spatial analysis, cancer informatics, histology image analysis, explainability

## Abstract

*Objective.* Histology image analysis is a crucial diagnostic step in staging and treatment planning, especially for cancerous lesions. With the increasing adoption of computational methods for image analysis, significant strides are being made to improve the performance metrics of image segmentation and classification frameworks. However, many developed frameworks effectively function as black boxes, granting minimal context to the decision-making process. Thus, there is a need to develop methods that offer reasonable discriminatory power and a biologically-informed intuition to the decision-making process. *Approach.* In this study, we utilized and modified a discriminative feature-based dictionary learning (DFDL) paradigm to generate a classification framework that allows for discrimination between two distinct clinical histologies. This framework allows us (i) to discriminate between 2 clinically distinct diseases or histologies and (ii) provides interpretable group-specific representative dictionary image patches, or ‘atoms’, generated during classifier training. This implementation is performed on multiplexed immunofluorescence images from two separate patient cohorts- a pancreatic cohort consisting of cancerous and non-cancerous tissues and a metastatic non-small cell lung cancer (mNSCLC) cohort of responders and non-responders to an immunotherapeutic treatment regimen. The analysis was done at both the image-level and subject-level. Five cell types were selected, namely, epithelial cells, cytotoxic lymphocytes, antigen presenting cells, HelperT cells, and T-regulatory cells, as our phenotypes of interest. *Results.* We showed that DFDL had significant discriminant capabilities for both the pancreatic pathologies cohort (subject-level AUC-0.8878) and the mNSCLC immunotherapy response cohort (subject-level AUC-0.7221). The secondary analysis also showed that more than 50% of the obtained dictionary atoms from the classifier contained biologically relevant information. *Significance.* Our method shows that the generated dictionary features can help distinguish patients presenting two different histologies with strong sensitivity and specificity metrics. These features allow for an additional layer of model interpretability, a highly desirable element in clinical applications for identifying novel biological phenomena.

## 1. Introduction

Histopathological image analysis of tissue specimens is one of the primary clinical methods used by physicians for disease diagnosis and monitoring (Madabhushi 2009). This is of prime importance in the diagnosis of various cancer pathologies. Owing to the advances in digital pathology, it is now possible to leverage computational tools to analyze and interpret tissue images to derive clinically relevant features. The advent of more informative and multiplexed imaging formats, such as immunohistochemistry-based imaging and cytometry by time-of-flight, has led to an increase in throughput, making it possible to image and analyze over 30 markers on average per slide (Bendall *et al* 2011, Tan *et al* 2020). For diseases such as cancer, there is a high degree of heterogeneity in the disease environment, from tissue to the genomic scale. A myriad of cells, both active and supportive in nature, are involved in cell signaling that influence disease progression, and their spatial arrangement offers insight into their inter-cellular dynamics (O’Connor *et al* 2014, Heindl *et al* 2015, Barua *et al* 2018). Moreover, imaging data generated from histology is often less expensive and more accessible in most diagnostic pipelines. This is supplemented by the possibility of deriving secondary image features that are highly correlated with features derived from more complex multi-omics data such as genomics and transcriptomics (Yuan *et al* 2012, Antonelli *et al* 2019). The availability of these features would allow for the full utilization of this rich dataset in low-resource settings. Thus, it would be prudent to derive image-based features that can identify and predict trends in the interaction between multiple cell types in a sound manner.

In recent years, the usage of dictionary learning-based methods for various medical image classification tasks has been increasing (Zhu *et al* 2018, Zhao *et al* 2019). In dictionary learning, class-specific features obtained from training data are used as dictionary ‘elements’, that are representative of a given class. Such methods allow for the representation of these features, or ‘signals’ using constituent elements or ‘bases’ learned from a dataset as opposed to defining them in advance, leading to an additional layer of personalization to the problem (Vu *et al* 2015). Dictionary-based methods are powerful in that they provide visual contexts and a layer of explainability through the availability of the dictionary ‘elements’, which allows for the identification of concurrent features across all members of a given disease cohort.

Dictionary learning integrated with sparse representation methods has proven to be powerful in increasing classification accuracy in multiple applications for image denoising and classification, and encouraging results have been observed in a variety of use cases (some examples being Aharon *et al* 2006, Srinivas *et al* 2013, Al-Shaikhli *et al* 2014, Cruz and Alfonso 2015, Barua *et al* 2016). Specifically, a variety of sparse representation-based frameworks have been proposed in the medical imaging sphere for the classification and quantification of histologically stained whole-slide images (Shirale 2018). For example, Romo *et al* (2014) proposed a multi-scale descriptor to describe specific elements in a histological slide, using dictionaries built separately for each element of the slide using sparse coding algorithms based on the obtained multi-scale visual elements. Similarly, Vu *et al* (2015) proposed and validated a low-complexity discriminative feature-oriented dictionary learning method on three real-world datasets, including brain tumor images obtained from TCGA. In these cases, image morphological and textural features were used to create curated class-specific dictionaries. These features can be affected by irregularities in staining across samples and other artifacts introduced during image acquisition, leading to a fair amount of investment of effort in feature engineering and curation. It would be useful to examine whether such sparse-representation dictionary learning methods can be applied to data where only positional and phenotyping information obtained from staining and imaging are utilized.

We begin our proposed idea by wanting to classify different types of images into their correct class of membership. One way to imagine an image is as a composite or a combination of one or multiple ‘bases’ or constituent elements/patterns. Many of these bases can be unique to a given class, and we want to attempt to identify these discriminative bases from each class for (1) classification purposes and (2) to analyze the structural and morphological features of these bases to understand the underlying biology.

In this study, we utilize a kernel-smoothed representation of spatial point patterns of various cell phenotypes identified in tissue multiplexed immunofluorescence (mIF) images to train a discriminative dictionary learning-based image classifier capable of identifying features used to design a class-specific dictionary. For our proof-of-concept study, we used a modified version of the discriminative feature-based dictionary learning (DFDL) algorithm proposed by Vu *et al* (2016) as the classifier of choice. We implemented our framework on two distinct cohorts, one of pancreatic malignancies (herewith referred to as PanC) and another of metastatic non-small cell lung cancer(mNSCLC) as described:

• The PanC dataset consists of two distinct pathologies, one non-cancerous(chronic pancreatitis(CP)) and one cancerous (pancreatic ductal adenocarcinoma (PDAC)). These two pathologies are known to share many stromal and cellular traits, which makes discrimination of the two often difficult.
• The mNSCLC dataset consists of mIF images from patients who received the immune checkpoint inhibitor (ICI) immunotherapy regimen and were responsive or non-responsive to the treatment. Work has been undergone in utilizing mIF images for assessing spatial relationships in the NSCLC space in the past few years, with work done by Surace *et al* (2020) and Barua *et al* (2018) as examples. Understanding the differences in inter-cellular relationships in the tumor microenvironment across these two cohorts in a more informed manner is crucial to developing more personalized treatment paradigms.

Broadly, our DFDL-based workflow entails the following steps:

(i) Generate a smoothed representation of the point pattern phenotypes of interest, and create a composite stack ‘image’ representation.
(ii) Divide the images from each class of the patient cohort into training and test sets, and obtain patches from each image.
(iii) Apply the dictionary learning framework to learn a dictionary for each class, where each dictionary is composed of atoms that well explain images from their own class and poorly explain images from the other classes.
(iv) Classify the images from the test set based on the generated class dictionaries.

**Figure 1.**
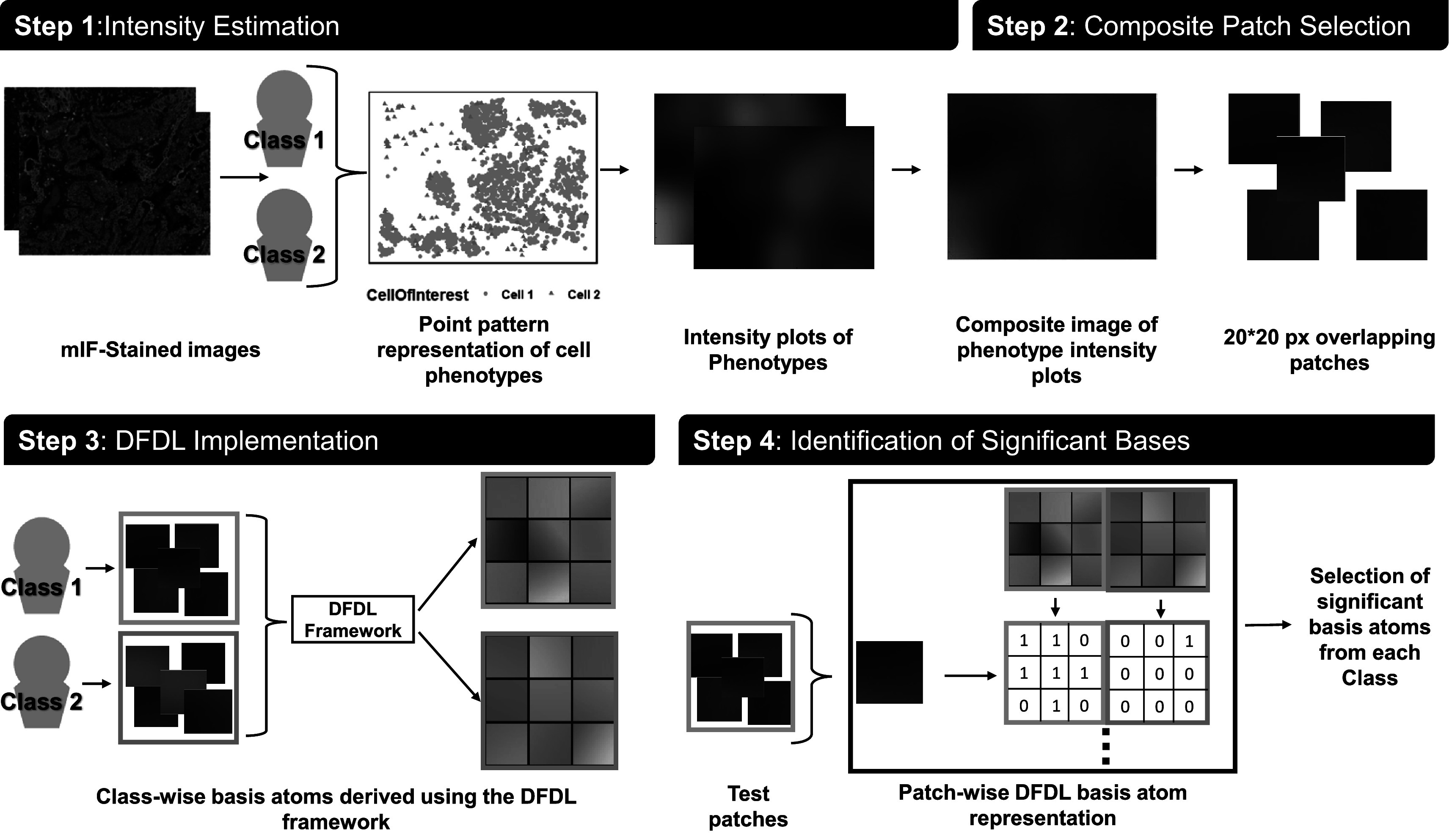
A schema of the proposed framework. The framework utilizes patches obtained from point intensity maps of the cell phenotypes under consideration. Representative patches from the two classes are then used as training inputs for our dictionary learning-based classifier to distinguish between any two classes of diseases. The generated dictionary elements or ‘atoms’ can then be utilized to generate a sparse representation of every image in the dataset as some combination of the representative atoms from each group.

A schematic summary of the framework implementation is presented in figure 1. To give a brief outlook of the paper, pertinent data and acquisition parameters are discussed in section 2.1. This is followed by an explanation of the preprocessing steps and a generalized look into the overall DFDL framework in sections 2.2–2.4. Data-specific preprocessing, classifier results, and results from a comparative analysis of the obtained dictionary atoms are highlighted in section 3. Finally, we discuss some of the biological interpretations of the results obtained and future improvements in section 4.

## 2. Materials and methods

In our framework, we employ the spatial point pattern of phenotypes of interest obtained from histology data analysis to generate a smoothed kernel representation of the same. For each image, we then used these smoothed kernel representations as input to (1) generate patches of interest, (2) train and test the dictionary learning framework on these patches, and (3) evaluate the obtained sparse dictionary atoms, or group-specific patches representing a specific class, for significant correlations across pairs of phenotypes in each cohort. In the subsequent sections, we explain the clinical data acquisition procedures and the aforementioned steps.

### 2.1. Clinical data

The study cohort consisted of mIF-stained images belonging to 2 different datasets: (a) a pancreatic cohort consisting of patients diagnosed with CP and PDAC and (b)an mNSCLC dataset consisting of patients who underwent a standard immunotherapeutic treatment regimen. The pancreatic cohort was obtained from patients at the University of Michigan Pancreatic Cancer Clinic. The mNSCLC cohort was obtained from patients from the University of Michigan treated with standard-of-care immunotherapy between 2015 and 2017. Patient data collection and retrieval were performed in accordance and approval of the University of Michigan's Institutional Review Board.

To phenotype tissue slides from patients with pancreatic disease, multiplexed immunofluorescence staining was performed on a tissue microarray composed of 0.6 mm cores taken from formalin-fixed paraffin-embedded (FFPE) tissue blocks, as explained and validated in previous works (Lazarus *et al* 2018, 2019). For the lung cancer patients, whole slide images were utilized. In both cohorts, the slides underwent serial rounds of antigen retrieval, followed by primary and secondary antibody staining. DAPI nuclear staining was performed to segment the nuclei and assign spatial locations to every cell identified. Cytoplasmic and nuclear fluorescence was used for phenotyping using antibodies to identify the expression of CD3, CD8, pancytokeratin, and FoxP3. Specific cell types identified included: the Epithelial cells (PanCK+), immunosuppressive regulatory T-cells (Treg: CD3+ CD8-, FoxP3+), immunoreactive Cytotoxic Lymphocytes (CTL: CD3+, CD8+), Antigen presenting Cells (APC: CD163+), and the HelperT-cells (HelperT: CD3+CD8-FoxP3-). All phenotyping and processing of the images were performed using AKOYA Biosciences’ Inform Software. Clinical and demographic information for the cohorts are presented in table 1.

**Table 1.** A summary of clinical characteristics of the patient cohort. Missing values were excluded when computing summary statistics in each category.

Characteristics		PanC cohort		mNSCLC cohort	
		CP	PDAC	Responders	Non-responders
Number of patients		*N* = 34	*N* = 71	*N* = 36	*N* = 27
Number of images		*N* = 56	*N* = 139	*N* = 700	*N* = 425
Average number of image slides per patient		1.64	2.01	19.4	15.75
Image pixel resolution		0.5 microns/pixel		1 micron/pixel	
Sex	Male	13	31	15	0
	Female	6	29	13	13
Smoking Status	Former/Current	17	33	33	21
	Never	4	26	3	6
	Unknown	13	12	0	0

### 2.2. Phenotype intensity-based composite image generation

#### 2.2.1. Cell phenotype intensity estimation

The nuclei staining and identification described in the previous section allow us to obtain the spatial locations of each cell, along with associated phenotypic information. It is possible to utilize this data to generate a visualization where each cell in the image can be thought of as a point in two-dimensional space, a collection of which is termed a spatial point pattern (Wiegand and Moloney 2014). Rather than using the point patterns in our analysis, we considered a kernel-smoothed intensity representation of each cell type as input to our data, the details of which are explained in Krishnan *et al* (2022).

Briefly, we computed a two-dimensional isotropic kernel intensity estimate for every point belonging to each cell-specific point pattern on a spatial grid. This is then used to generate a point-intensity image for each cell type of interest. Care is also taken to ensure that the spatial grid is proportional to the size of the original slide and edge effect bias is accounted for in the computed intensity grid (Diggle 1985). Computations were performed using the *spatstat* (Baddeley and Turner 2005) package in R software (R Core Team 2013). Through this process, we obtain a number of intensity surfaces, with each of the surfaces corresponding to the phenotypes considered in the study. A visual representation of the smoothed intensity surfaces for one of the cell types from one representative image is shown in supplementary figure 1.

#### 2.2.2. Composite patch selection

It would be effective to combine the intensity surfaces and utilize them as inputs for the DFDL framework in a joint manner. To this end, the individual intensity surfaces associated with a given image are stacked to form a three-dimensional matrix array representation. Each image is of the size *l* × *m* × *c*, where *l* and *m* correspond to the dimensions of the original point pattern intensity image obtained, and *c* to the number of phenotypes or ‘colors’ considered, analogous to a typical RGB image structure. Through this process, we obtain a pseudocolor representation of the distribution of each cell phenotype in a given image.

For the initialization of the dictionary learning framework, rather than use the complete generated composite image as input, we use smaller patches extracted from the image as input so as to be able to capture the difference in interaction characteristics across sub-geographies of the image. For this purpose, we extract *n* × *n* × *c* square pixel patches from each image from each of the patient classes under consideration. These patches can either be sequentially or randomly extracted and can be overlapping or not, depending on the particular use case. After extraction, they undergo an additional elimination step, where extremely low-intensity-valued patches(with more than 70% of the patch pixel intensities less than 10^−15^) are removed from consideration. Once these patches have been prepared, we initialize the DFDL framework to train the classifier and obtain class-specific dictionaries.

### 2.3. Implementation of the DFDL framework

The discriminative feature-oriented dictionary learning (DFDL), proposed by Vu *et al* has previously been applied to histopathological image classification tasks. An offshoot of the dictionary learning methods commonly used in machine learning applications, this method allows for the creation of class-specific dictionaries that maximizes intra-class differences while minimizing inter-class differences (Vu *et al* 2015). In other words, the features learned from DFDL are such that,

• The learned features explain images from their own class well
• The learned features explain images from the other class poorly

DFDL solves the optimization problem of finding these visual features, which we address as dictionary atoms. The basic principles of DFDL pertinent to our implementation are explained below.

#### 2.3.1. Training data curation

**Figure 2.**
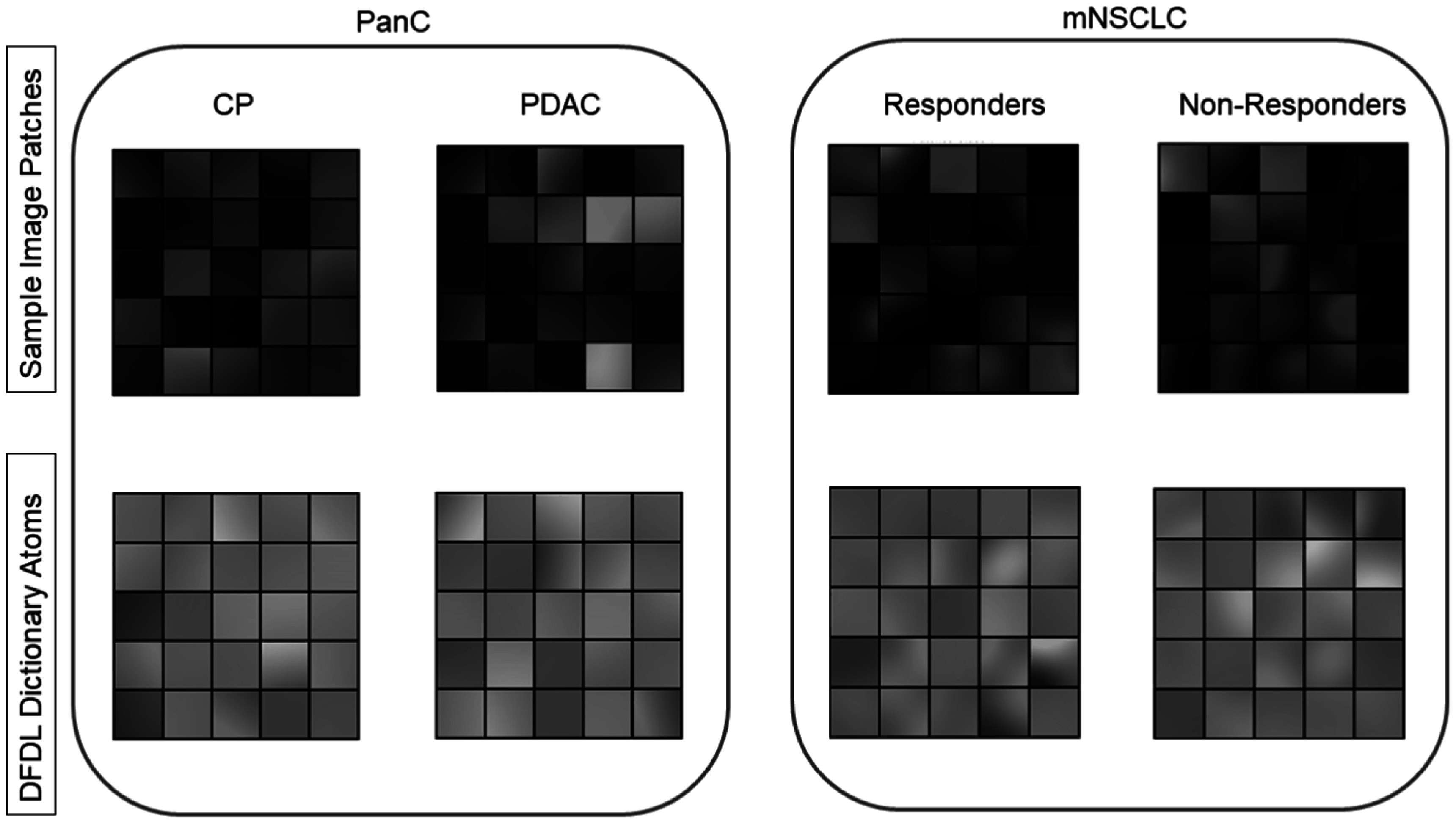
A representation of the image patches used as input for the DFDL framework. It is important to note that only 3 out of the possible 5 surfaces have been displayed, due to image display constraints with more than 3 color surfaces.

We select a number of patches proportional to each class size considered for training. Each sample image patch is vectorized from its *n* × *n* × *c* structure to a 1-dimensional vector, represented as *p*. Because this is a binary classification problem, we consider two classes, *Y* and $\bar{Y}$, with their constituent *ith* and *jth* patches represented as *p*_*Yi*_ and ${p}_{\bar{{Yj}}}$, respectively. These restructured patches are fed into the DFDL algorithm. Figure 2 depicts a visualization of a sample of the input image patches generated, as mentioned in the previous section, and the corresponding dictionary atoms obtained for each of the classes in both our clinical cohorts.

#### 2.3.2. Implementation and training of the dictionary learning framework

When implementing the framework, we use a LASSO+OMP approach to find the best set of dictionary atoms (SPAMS: SPArse Modeling Software, n.d.). Equation (1) summarizes the optimization algorithm:

\begin{eqnarray*}{D}^{* }=\arg \mathop{\min }\limits_{D}\left(\displaystyle \frac{1}{N}\mathop{\min }\limits_{\parallel S{\parallel }_{0}\leqslant L}{\parallel Y-{DS}\parallel }_{F}^{2}-\displaystyle \frac{\rho }{N}\mathop{\min }\limits_{\parallel S{\parallel }_{0}\leqslant L}{\parallel \bar{Y}-D\bar{S}\parallel }_{F}^{2}\right).\end{eqnarray*}

For the patches of each class *Y* and $\bar{Y}$, we learn a dictionary *D*_*i*_, *i* = (1, 2) for each class, where *D*_1_ corresponds to the dictionary for Class 1, and *D*_2_ for Class 2 in a binary classiciation problem. These learned dictionaries try to explain patches from their own class well while being poor at explaining patches from another class. The dictionaries are learned with a sparsity constraint *kSk*_0_ ≤ *L*, where *S* is a vector with at most *L* nonzero elements. More details on the choice of this sparsity parameter, *L*, is given in the supplementary section. The parameter *ρ* is a trade-off parameter. A low *ρ* drives the algorithm to learn a *D*_*i*_ that is very good at explaining patches from its own class but might not be poor at explaining patches from the other class. A high *ρ* has the opposite effect: the algorithm tries to learn a *D* that is very poor at explaining patches from the other class, but is also not good at explaining patches from its class.

#### 2.3.3. Patch-level classification scheme

First, we compute a class label for each patch. We then combine the patch-level predictions for all patches using a voting scheme to generate a class label for the entire image. We concatenate dictionaries *D*_1_ and *D*_2_ to form a combined dictionary, *D*_combined_. We then compute the sparse representation *S*_test_ of a given *Y*_test_ using *D*_combined_. We split *S*_test_ into constituent sub-representations *S*_test,1_ and *S*_test,2_ for each class. Then we compute the corresponding errors for each class as shown in equations (2) and (3)

\begin{eqnarray*}{\epsilon }_{1}={Y}_{{\mathrm{test}}}-{D}_{1}{S}_{{\mathrm{test}},1.}\end{eqnarray*}

\begin{eqnarray*}{\epsilon }_{2}={Y}_{{\mathrm{test}}}-{D}_{2}{S}_{{\mathrm{test}},2}.\end{eqnarray*}

The assigned patch label is the class with the smaller error. If ϵ_ 1_ is smaller, then the assigned patch label is 1; otherwise, it is 2.

#### 2.3.4. Image and subject level classification scheme

For a given image or patient, we determine the class labels for each constituent patch. We compute a ratio θ , which is given as the ratio of the number of patches predicted as class 1(our reference class) to the total number of patches for that image or subject. This computed θ can be used as a probabilistic representation of class membership in computing performance metrics. This can be represented as follows:

\begin{eqnarray*}\theta =\displaystyle \frac{N{\left({\mathrm{patches}}\right)}_{{\mathrm{class}}1}}{N{\left({\mathrm{patches}}\right)}_{{\mathrm{total}}}}.\end{eqnarray*}

The implementation of the composite image preprocessing, the DFDL framework, and the computation of the performance metrics were done on MATLAB (MATLAB version 9.5.0.1298439 (R2018b), 2018).

### 2.4. Identification and analysis of significant dictionary atoms

Following the implementation of the framework, we want to further assess the validity of biologically relevant information captured by the atoms that were identified as significantly discriminant according to the DFDL framework. Given that DFDL allows us to emphasize in-group over out-group differences through the selection of an appropriate *ρ*, it is reasonable to assume that the representative dictionary elements also capture interactions that distinctly belong to one of the two classes only. Since we want to analyze the more informative bases, we select highly active atoms across each group in the cohort.

For this purpose, we compute the sparse code, *S*, for every patch for each image in our training and test sets, with *S* being a vector of the same length as the total number of dictionary atoms across both classes obtained through the patient-wise analysis, *D*_combined_. Since we want to analyze the constituent atoms of each group separately, we only pick up that portion of the image code that represents the true class to which the image/patient belongs. Hence, for a *D*_combined_ of length *l*, we consider only the first $\tfrac{l}{2}$ elements of the code for Class 1 called *S*_1_. Similarly, for images from Class 2, we consider only the last $\tfrac{l}{2}$ elements of their code as *S*_2_. We then normalize individual sparse code values associated with each atom across all image patches. Based on the value and direction of each code, we re-code them in the following manner:

\begin{eqnarray*}{A}_{i}=\left\{\begin{array}{ll}1 &amp; \mathrm{for}\quad {A}_{o}> 0\\ 0 &amp; \mathrm{otherwise}\end{array}\right.,\end{eqnarray*}

where *A*_*i*_ is the new binary code with 1 representing a positive sparse code value for a given representative base and 0 if the sparse code value is either zero or negative, and *A*_*o*_ is the original sparse code value. Negative values were excluded since they describe poorly represented bases in the image. We now utilize the re-coded values to compute the proportion of all atoms (atom dictionary *D*_1_ for Class 1, and *D*_2_ for Class 2) separately. A paired t-test is performed to compare the change in proportions across the cohorts. The sign and magnitude of the t-values obtained are used to identify the relative significance of each representative atom in each cohort.

After identifying the significant atoms from each class using the t-test values and their corresponding *p*-value (significance at *p* < 0.05), we further explore the relationships between the different cell types that constitute the composite image used as input to our framework. For this purpose, we perform a spatially weighed partial correlation analysis for each pair of cell phenotype surfaces of the class atoms under consideration. This process was repeated individually for every dictionary atom obtained from the framework. Partial correlation analysis is useful for quantifying the correlation between any two variables, or in our case, any two cell phenotypes, while accounting for the confounding effect of other phenotypes not under consideration (Whittaker 2009, Everitt and Skrondal 2011). A uniformly varying Gaussian kernel defined as :

\begin{eqnarray*}k({x}_{i},{x}_{j})=\exp \left(\displaystyle \frac{-{\parallel {x}_{i}-{x}_{j}\parallel }^{2}}{{\sigma }^{2}}\right),\end{eqnarray*}

was used as the weighting factor *W* in the computation of the covariance matrix used in the correlation analysis, where $(\parallel {x}_{i}-{x}_{j}\parallel )$ is the Euclidean distance given by

\begin{eqnarray*}\parallel {x}_{i}-{x}_{j}\parallel ={\left({\left({x}_{i}1-{x}_{j}1\right)}^{2}+{\left({x}_{i}2-{x}_{j}2\right)}^{2}+...+{\left({x}_{i}D-{x}_{j}D\right)}^{2}\right)}^{.}5,\end{eqnarray*}

and *σ* ^2^ is the bandwidth of the kernel (Hainmueller and Hazlett 2017). This is repeated for all the dictionary atoms under consideration. The proportion of significantly correlated intensity surface pairs is then computed separately for each group in the cohort. Associated correlation analysis was performed using the *corpcor* (Schäfer, n.d.), *tseries* (Trapletti and Hornik 2022), *stats*, and *pracma* (Borchers 2021) packages in *R* (R Core Team 2020).

## 3. Results

We applied the modified DFDL framework to two different datasets: a pancreatic cohort consisting of patients with CP or PDAC, and a lung dataset with patients who responded and did not respond well to an ICI immunotherapeutic regimen. The framework was implemented separately at both the image and subject levels. In the patient-wise analysis scenario, we treat each patient as an individual sample, and group images together such that images associated with a patient who is part of the training set are not found in the testing set, and vice versa. In the image-wise analysis, we treat each individual image as a sample and disregard patient-image associations. This was done as it was observed that some patients had multiple associated images; this is highly notable in the case of the mNSCLC dataset, with an average of 20 images associated with each patient, as opposed to the PanC data, where most patients had only one. Factoring this variance into our workflow, it was of interest to see how much of a disparity, if any, would be observed in the classifier performance in these two scenarios. Further details on data-specific preprocessing and the results are described below.

### 3.1. Application on the pancreatic dataset

In our analyses, we utilized point pattern data extracted from multiplexed immunofluorescence images belonging to two distinct pancreatic disease pathologies which share many histological traits. Phenotype surfaces corresponding to five phenotypes of interest, namely, epithelial, CTL, HelperT, Treg, and APC were used to generate the composite image. The DFDL framework looks for regions of significance that are representative of a given group across all input images, with each image component contributing jointly to the dictionary generation process. Thus, we generate a 70 × 50 × 5 pixel composite image from the intensity surfaces, with each pixel corresponding to an actual slide area of 25 *μ*m^2^. Next, 20 × 20 × 5 overlapping patches were extracted from each composite image. The dimensions of the patches were selected to ensure that no macroscopic interactions were missed by making them too small, and no interesting local patterns were missed by making them too large. Additionally, based on the recommendations from the pathologist, we extracted patches with a reasonable overlap between them. An intermediary filtration step is inserted to ensure patches with very low intensity values were dropped across all cell intensity surfaces . Each individual three-dimensional image tensor was then vectorized to a 2000 × 1 matrix to create the desired input structure for the DFDL framework. For our initial analysis, we select 100 as the total number of dictionary elements or atoms, *D*_combined_, to be computed using the training paradigm, with 50 each for CP and PDAC.

#### 3.1.1. Classification results

5-fold cross-validation with an 80–20 train-test split on the data set was done for both classes under consideration to assess discriminator performance. The 5-fold image-level and patient-level area under the curve (AUC) metric, a standard metric for measuring classifier performance, with their respective sensitivities and specificities, are presented in table 2 (Bradley 1997). As we can observe, the image-level classifier (AUC = 0.8878) performs better than the subject-level classifier (AUC = 0.8217), although both of them have a reasonably impressive performance with high specificity with reasonable sensitivity metrics. We surmise that the high variability in the sample size between the 2 classes(CP = 56, PDAC = 139) may have influenced the observed lower sensitivity. As mentioned earlier, a small number of patients have multiple image slides included. The presence of images from a given patient in both the training and testing folds, can also explain the higher performance of the image-level classification paradigm. However, the higher AUC and specificity indicate good discriminatory behavior with a lower rate of false positive flagging, which is explained by the framework’s focus on deriving atoms that are representative of each group.

**Table 2.** The *n*-fold Area Under Curve (AUC) values obtained using the modified DFDL on the data set in an image-wise and subject-wise manner, along with the associated sensitivities and specificities. The algorithm is trained on a proportional number of patches from each class, with each cohort divided into 5 folds. The best results were obtained when 50-atom dictionaries were learned for each of the two classes (CP and PDAC).

Patient	Number of folds	Number of atoms	Atom size	AUC	Sensitivity	Specificity
Cohort	(k)	per group	(pixels)	(k-fold CI)	(k-fold CI)	(k-fold CI)
Pancreatic Cohort	5	50	20	0.8217	0.625	0.9319
(Patient-wise)				(0.7–0.9048)	(0.5–0.875)	(0.8095–1)
Pancreatic Cohort	5	50	20	0.8878	0.5743	0.9562
(Image-wise)				(0.7828–0.9996)	(0.3636–0.8182)	(0.9259–1)

#### 3.1.2. Dictionary atom analysis

**Figure 3.**
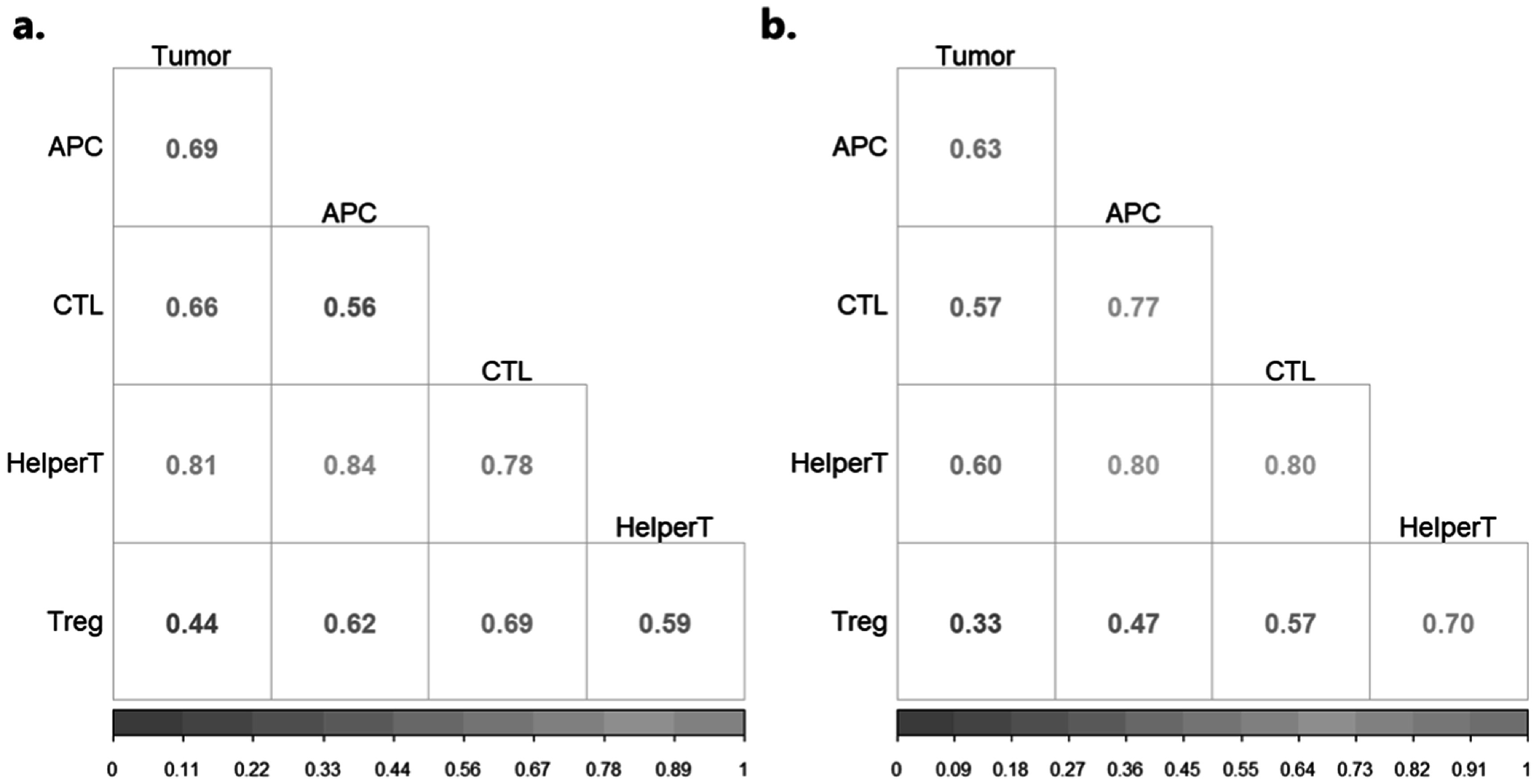
A modified proportions plot showing the proportion of total dictionary atoms (*N* = 50) showing significant partial correlation values between any given pairs of cell intensity surfaces, with (a) representing CP dictionary atoms and (b) representing PDAC dictionary atoms. It is observed that more than 50% of the atoms identified by the DFDL classifier show some correlation between various cell surfaces. This shows that the identified atoms carry information biologically relevant to the disease class . More information, including the corresponding raw significant atom counts, is presented in supplementary table 1.

In addition to classification, the representative class bases obtained from the DFDL training step can be used to study the underlying biological interactionsrecurrent in a significant portion of the cohort. From our framework, we obtained 100 dictionary atoms, with 50 each representative of CP and PDAC Using the methodology outlined in section 2.4, we selected the most active and significantly discriminative atoms from the CP and PDAC dictionaries, with 32 and 30 atoms identified for each class, respectively. We then ran a partial correlation framework to identify the proportion of atoms in which a given phenotype pair was found to be correlated(*p* < 0.05). The corresponding phenotype pairs and proportions are presented pictorially in figure 3 and in supplementary table 1. With the exception of a few pairwise analyses, namely Treg-Tumor for CP, Treg-Tumor, and Treg-APC for PDAC, more than 50% of the atoms displayed a strong correlation between any two given cell types.

### 3.2. Application on the lung dataset

Similar to the pancreatic dataset, we utilized point pattern data extracted from multiplexed immunofluorescence images belonging to mNSCLC patients showing differing responses to a standard immunotherapy regimen. Similar phenotypic intensity surfaces, as used in section 3.1 are also utilized for this data. No exclusion criterion was applied to this data set. Again, we generate a 70 × 50 × 5 pixel composite image from these 5 intensity surfaces, with each pixel corresponding to an actual slide area of 104 *μ*m^2^. Next, 20 × 20 × 5 overlapping patches were extracted from each composite image across both cohorts. Each individual three-dimensional image tensor was vectorized to a 2000 x 1 matrix and concatenated column-wise to create the desired input structure for the DFDL framework. Similarly, we select 100 as the total number of dictionary elements *D*_combined_, or atoms, to be computed using the training paradigm, with 50 each for responders and non-responders.

#### 3.2.1. Classification results

*N*-fold cross-validation on an 80–20 train-test split of the data set was done for both classes under consideration to assess discriminator performance. The 5-fold image-level and 4-fold patient-level classifier AUC, sensitivity and specificity are presented in table 3 (Bradley 1997). In this case, we observe the image-level classifier (AUC = 0.7176) performs marginally better than the subject-level classifier (AUC = 0.7057), with comparable specificity and high sensitivity metrics across all folds. Though the observed AUC and specificity metrics are lower as compared to the PanC cohort, the sensitivity for both the subject-level(0.7386, CI = (0.6714–0.8714)) and image-level (0.8334, CI = (0.7778–1)) are high enough to ensure accurate categorization of images to their true class. In contrast to the PanC cohort, the observed consistency in the classification metrics between the image-level and subject-level analysis points to a minimal effect of the train-test splitting paradigm on results in similar datasets.

**Table 3.** The *n*-fold area under the curve (AUC) values obtained using the modified DFDL on the data set in an image-wise and subject-wise manner, along with the associated sensitivities and specificities. The algorithm is trained on a proportional number of patches from each class, with each cohort divided into 5 folds (4 in the case of the subjectwise analysis). The best results were obtained when 50-atom dictionaries were learned for each of the two classes (Responders and Non-Responders).

Patient	Number of	Number of atoms	Atom size	AUC	Sensitivity	Specificity
Cohort	folds(k)	per group	(pixels)	(k-fold CI)	(k-fold CI)	(k-fold CI)
mNSCLC Cohort	4	50	20	0.7057	0.7386	0.6024
(Patient-wise)				(0.6524–0.7562)	(0.6714–0.8714)	(0.3176–0.7529)
mNSCLC Cohort	5	50	20	0.7176	0.8334	0.6607
(Image-wise)				(0.6032–0.8254)	(0.7778–1)	(0.5–0.8571)

#### 3.2.2. Dictionary atom analysis

**Figure 4.**
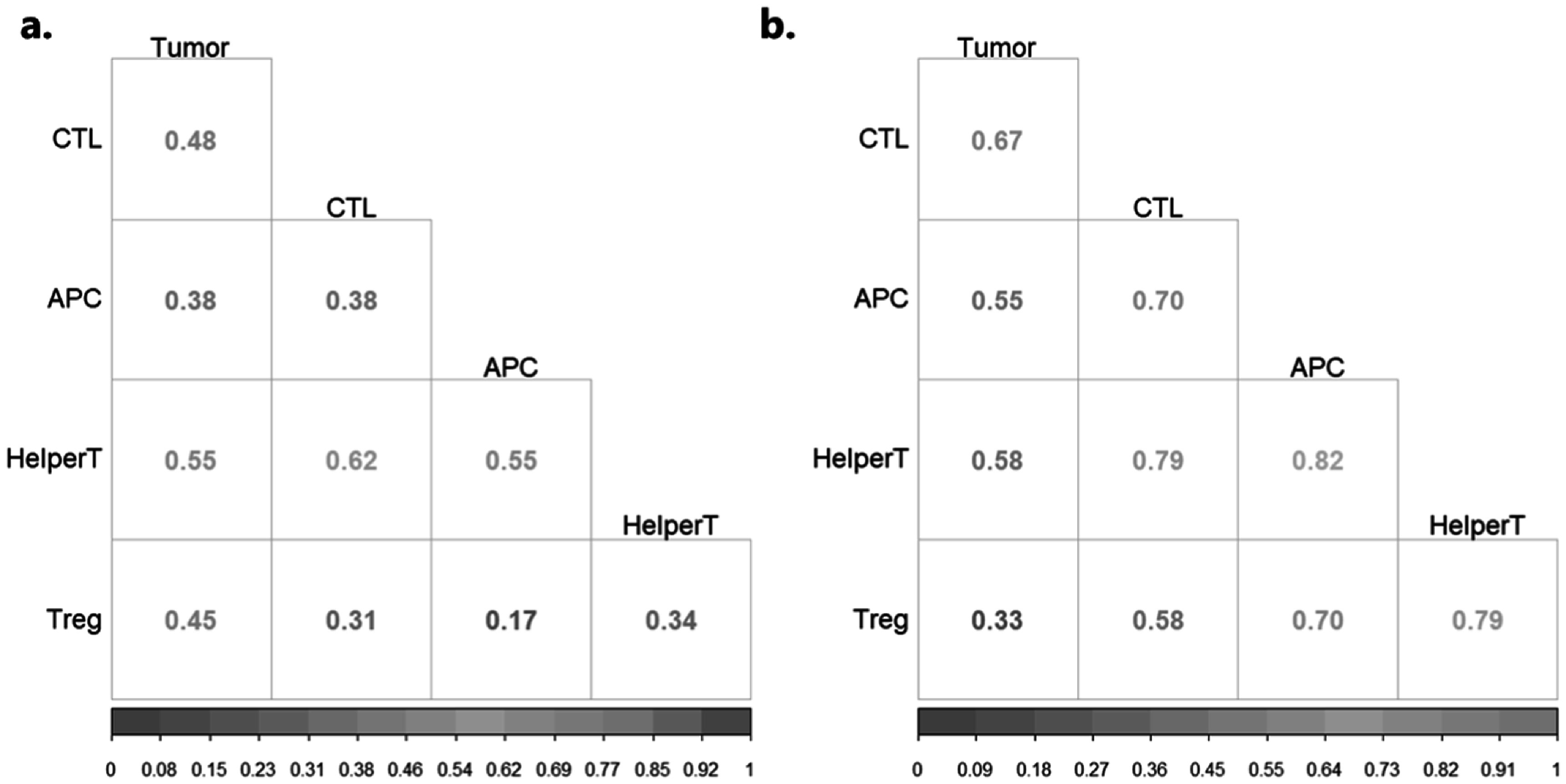
A modified proportions plot showing the proportion of total dictionary atoms (*N* = 50) showing significant partial correlation values between any given pairs of cell intensity surfaces, with (a) representing the significant dictionary atoms from the Responders cohort and (b) representing the significant dictionary atoms from the Non-Responders cohort. It is observed that a significant proportion of the atoms identified by the DFDL classifier show some correlation between various cell surfaces. This indicates that the identified atoms do carry information biologically relevant to the disease class considered. More detailed information, including the corresponding raw counts, is presented in supplementary table 2.

Similar to the PanC dataset, we used the methodology outlined in section 2.4 to select the most active and discriminatory atoms across both classes in the cohort. Of the 100 total dictionary atoms obtained, we identified 29 and 33 atoms from the Responders and Non-Responders classes, respectively. Again, a partial correlation framework was run to identify the proportion of atoms in which a given phenotype pair was found to be correlated (*p* < 0.05). The corresponding phenotype pairs and proportions are presented pictorially in figure 4 and tabulated in supplementary table 2. In this case, it is observed that a smaller set of phenotype pairs are correlated among at least 50% of the significant atoms representing Responders, as opposed to the Non-responders.

## 4. Discussion

In our study, we explored the idea of discriminative dictionary learning to discern between different disease pathologies in a visually interpretable manner. Though deep-learning-based approaches have been explored to analyze mIF images in a spatially salient manner (for example, in work done by Fassler *et al* (2020)), in our approach, we utilize an alternate joint representation of the point pattern data extracted from the stained and processed images as inputs to our framework. As mentioned earlier, the framework looks for regions of significance that are representative of a given group, from either real RGB or grayscale images, with each component contributing jointly to the dictionary generation process. Thus, this setup allowed us to analyze the generated discriminatory atoms from each group, and identify biologically-informed relationships between the different cell types demarcated in each case.

As stated earlier, we did both an image-wise and patient-wise analysis to compare and contrast performance across these two different methods of defining training and testing samples. It is important to note that there is a potential for data leakage in the image-wise analysis, as the treatment of each image as a separate sample can mean that images belonging to the same patient end up both in the training and validation set. Thus, it is reasonable to conclude that though we have results from both analysis paradigms, from an interpretation standpoint, the patient-wise results for both the lung and pancreatic datasets are more robust.

We wanted to further analyze the dictionary atoms found to be relevant to a given clinical histology to assess the legitimacy of the atoms captured. It would be interesting to compute the correlations between the constituent phenotype surfaces, and see what proportion of correlated surfaces were enriched or depleted in one class over the other. To this end, we performed a 2-sample z-test between the proportions of correlated phenotype pairs from the two classes, with the results presented in supplementary tables 1 and 2.

Discrimination of PDAC and CP remains a significant pathologic conundrum leading to misdiagnosis and unnecessary re-biopsy and occasionally incorrect treatment. Each pathology has an accumulation of inflammatory and suppressive immune cells, which differ slightly in proportion and spatial distribution. We attempted to use a DFDL framework to harness subtle differences in immune cell infiltration and spatial relationship to more accurately determine diagnosis. It is observed that the development of the pancreatic tumor microenvironment is accompanied by a turn toward an immunosuppressive environment (Foucher *et al* 2018). It is marked by an accumulation of T-regulatory and other HelperT phenotypes, often with protumor function. Consistent with this, our data demonstrate the number of atoms showing a significant correlation between HelperTs and Tumor cells is lowered in PDAC as opposed to CP (*p*-val = 0.0329) (Halim *et al* 2017).

In addition to aiding in pathologic diagnosis, we sought to determine if the DFDL framework could be used to predict response to therapy. Immune checkpoint inhibitors against PD-1/PD-L1 are being increasingly adopted as prime treatment measures against metastatic NSCLC, either following chemotherapeutic treatment or in an adjuvant context (Reck *et al* 2016, Gandhi *et al* 2018). A closer examination of the disease microenvironment through spatially-aware biomarker expression methods after the therapeutic administration is crucial to ensuring optimal treatment response (Fu *et al* 2021). Currently, there are no good biomarkers to predict response to therapy, with most relying on PD-L1 mono-staining. We hypothesized that the presence of spatial relationships between immune and epithelial cells in the lung cancer tumor microenvironment could better characterize endogenous immune reactivity, which is paramount to the success of checkpoint inhibitor-based therapy (Altorki *et al* 2018). Through our analysis, we observed that there is a significant increase in Treg engagement with CTLs, APCs, and HelperT cells in non-responders as opposed to responders(shown in supplementary table 2). Additionally, there is an increase in the correlation between APCs with CTLs and HelperT cells. These results show that DFDL could potentially use these unique relationships to identify patients more likely to respond to therapy.

From these results, we can surmise that the dictionary atoms are representative of the complexity of the data, and can be used to build representation models for different classes of diseases. The high AUC values in both cohorts (PanC Image-level—0.8878 and mNSCLC Image-level—0.7176) are indicative of the discriminative ability of the atoms generated. We also find that more than 50% of the atoms identified for each of the classes were found to be significantly represented in images from their respective classes, which points to the atoms being biologically meaningful. Finally, among the significant atoms, a sizeable fraction of them were shown to exhibit hallmarks of disease biology. Thus, we can conclude that the atoms identified from the DFDL framework are legitimate and biologically meaningful.

It is important to acknowledge that we cannot deduce the nature of the relationship between the different pairs of phenotypes identified as significant at this stage. A more rigorous analysis of each atom using spatially informed methods such as the G-function or GaWRDenMap framework would be needed to assess the strength and nature of the relationships identified to be significantly represented (Barua *et al* 2018, Krishnan *et al* 2022). In its current form, our framework only utilizes spatial and binary phenotypic data to create the surfaces with excellent classification metrics. It is possible, however, to integrate imaging data with associated genomic information as input to the framework and obtain higher classifier performance with more informative dictionary atoms for each group.

Our proposed framework attempts to utilize the dual strengths of dictionary learning techniques: classification power, and visually interpretable outputs, and is by no means comprehensive. As an extension to this work, we would like to explore other powerful dictionary learning algorithms, such as FDDL (Vu and Monga 2017) and DLSI (Ramirez *et al* 2010), and apply them in the context of our work. Because a significant fraction of the patches from either class of patients is similar, we believe that designing an additional dictionary will improve our results. That is, learning three dictionaries (*D*_1_, *D*_2_, and *D*_common_) will benefit the classification performance by reducing the confusion caused by similar patches and identifying features that are much more distinctive. Finally, it is also possible to extend the framework and learn dictionaries at multiple resolutions, thus helping us to find discriminative features at a coarser scale, such as nuclear arrangement and density (Romo *et al* 2014). However, it is also important to acknowledge that in its current form, additional considerations have to be taken into account before the deployment of our framework in the clinical setting. There are trade-offs in the sensitivity and specificity characteristics of a model based on a lot of factors, including the application space of the framework. Thus, a thorough clinical validation of our analysis metric, including error and uncertainty quantification, is needed before being pushed into clinical deployment.

In conclusion, we proposed a method to develop and train a dictionary-learning-based classifier to differentiate between two classes using a visual interpretable component. The generated dictionaries were able to satisfactorily differentiate between samples exhibiting different histologies of clinical importance. Additionally, the generated dictionary elements provide a way to query trends in interactions that are definitive for a given group, which might shed light on novel cellular dynamics or reconfirm previously existing ones. The additional insight facilitated by the DFDL framework can, in turn, assist in the development of better management strategies for people afflicted with various cancerous pathologies, including pancreatic and lung malignancies.

## Ethics statement

Studies involving human participants were reviewed and approved by the University of Michigan’s Institutional Review Board. The research was conducted in accordance with the principles embodied in the Declaration of Helsinki and in accordance with local statutory requirements. The patients/participants provided written informed consent to participate in the study. The associated approval IDs are HUM00098128 and HUM00157479.

## Funding statement

AR and SK were supported by CCSG Bioinformatics Shared Resource 5 P30 CA046592, a gift from Agilent technologies, and a Precision Health Investigator award from U-M Precision Health to AR along with L Rozek and M Sartor. SK and AR were partially supported by the NCI Grant R37-CA214955. SK and AR were also partially supported by The University of Michigan (U-M) startup institutional research funds. SK, SB, and AR were also supported by a Research Scholar Grant from the American Cancer Society (RSG-16-005-01). SB was supported by the NCI Cancer Center Support Grant NCI P30 CA016672, CPRIT RP110532, Center for Radiation Oncology Research Pilot Grant, NIH NCI U01 (CA196403), Institutional research grant from The University of Texas MD Anderson Cancer Center, Career Development Award from the Brain Tumor SPORE P50CA127001-07 and Equipment grant from NVidia. TL Frankel was funded by NIH Grant R01DK128120201.

## Conflict of interest declaration

AR serves as a member of Voxel analytics LLC and consults for Genophyll, LLC. SK, SB, and TF declare no potential conflict of interest.
